# Visible Helmet Presence Among Motorcycle Riders and Passengers at a Thai University: A CCTV-Based Cross-Sectional Observational Study

**DOI:** 10.3390/ijerph23050650

**Published:** 2026-05-13

**Authors:** Parkpoom Chaisiriprasert, Manaporn Chatchumni, Nuengruthai Petmeedee, Duangnapha Bunsong, Wassana Chaeypinij, Tanakon Asasing, Uma Khumwong, Nitthanet Natthakunlanan

**Affiliations:** 1 College of Digital Innovation, Rangsit University, Pathumthani 12000, Thailand; Parkpoom.c@rsu.ac.th (P.C.); nitthanet.n68@rsu.ac.th (N.N.); 2 School of Nursing, Rangsit University, Pathumthani 12000, Thailand; nuengruthai.p@rsu.ac.th (N.P.); duangnapha.b@rsu.ac.th (D.B.); 3 Health Welfare Office, Rangsit University, Pathumthani 12000, Thailand; wassana.c@rsu.ac.th (W.C.); uma.k@rsu.ac.th (U.K.); 4 Rangsit University Police Office, Rangsit University, Pathumthani 12000, Thailand; tanakon.a@rsu.ac.th

**Keywords:** visible helmet presence, motorcycle safety, road safety, university students, CCTV observation, Thailand

## Abstract

**Highlights:**

**Public health relevance—How does this work relate to a public health issue?**

- Motorcycle-related injury remains an important public health problem in Thailand, and low visible helmet presence in university settings increases preventable risk among young riders and passengers.
- This study addresses that issue by using real-world CCTV observation to assess actual helmet-wearing behavior during routine campus travel rather than relying on self-reported data.

**Public health significance—Why is this work of significance to public health?**

- Visible helmet presence was low overall (18.8%) across 19,363 motorcycle-user observation events, with extremely low compliance among student passengers (3.1%), identifying a particularly vulnerable group.
- The findings show that low visible helmet presence is patterned by role, affiliation, and time of day, indicating that campus road-safety risk is not uniform and can be more precisely targeted.

**Public health implications—What are the key implications or messages for practitioners, policy makers and/or researchers in public health?**

- Universities should be treated as priority settings for road-safety intervention, with strategies that specifically include passengers, student-targeted messaging, and evening-period risk reduction.
- Routine CCTV systems can serve as ethical, practical public health surveillance tools to identify high-risk groups, monitor behavior, and support evidence-based injury-prevention policy on campus.

**Abstract:**

Motorcycle-related injuries remain a major public health concern in Thailand, particularly among adolescents and young adults, yet evidence on actual helmet-related behavior in university settings remains limited. This study assessed visible helmet presence among motorcycle riders and passengers at a Thai university using real-world CCTV observations. A cross-sectional observational study was conducted at a large private university in Thailand using CCTV footage from two campus gates over seven consecutive days during peak commuting periods (07:00–09:00 and 16:00–18:00). Motorcycle-user observation events were coded for visible helmet presence, rider/passenger role, university affiliation, and time of observation. Descriptive statistics, chi-square tests, and 95% confidence intervals were used. A total of 19,363 motorcycle-user observation events were recorded. Overall visible helmet presence was 18.8% (3646/19,363). Visible helmet presence was 21.8% (1956/8985) among student riders, 3.1% (230/7505) among student passengers, 58.6% (1299/2217) among staff riders, and 24.5% (161/656) among staff passengers. Morning observation periods showed higher values than evening periods across most groups. Visible helmet presence was low overall and especially low among student passengers. These findings should be interpreted as event-based estimates rather than person-based prevalence or verified protective helmet use, and they support universities as priority settings for targeted road-safety interventions.

## 1. Introduction

Road traffic injuries remain a major cause of death and disability among adolescents and young adults worldwide, and motorcyclists bear a disproportionate share of this burden. In Thailand, motorcycles are widely used for daily travel and continue to contribute substantially to the national burden of road traffic injury despite longstanding helmet legislation and repeated road-safety campaigns [1,2,3,4,5,6,7,8,9,10,11,12,13,14,15].

Helmet wearing is one of the most effective protective behaviors for reducing the severity of motorcycle-related injuries. Evidence from a Cochrane systematic review showed that helmet use substantially reduces the risk of head injury and death among motorcycle riders involved in crashes [6,8]. In Thailand, enforcement of helmet legislation has previously been associated with improved helmet use and reduced traumatic head injury among motorcyclists [4]. Nevertheless, helmet-wearing compliance remains suboptimal, particularly among passengers. National evidence has shown a marked disparity between riders and passengers, with passengers consistently demonstrating lower helmet-wearing rates than drivers [13]. This gap suggests that the existence of helmet laws alone is insufficient to ensure consistent protective behavior across all road users.

University campuses are important microenvironments for road-safety research and intervention. In Thailand, many students and university personnel rely on motorcycles for commuting to and from campus and for short-distance travel within surrounding areas. Such routine travel may create a false sense of safety because journeys are often perceived as short, familiar, and low risk. Previous studies have shown that helmet-wearing behavior is influenced by multiple factors, including perceived susceptibility to injury, subjective norms, behavioral control, peer influence, and awareness of traffic law enforcement [1,5,14]. Young adults, especially passengers, often demonstrate lower compliance than riders, indicating an important behavioral and public health gap.

Although motorcycle safety has been studied in Thai university populations, much of the existing literature relies on self-reported survey methods. Such data are useful for understanding attitudes and intentions, but they may overestimate actual safety practices because of recall bias and social desirability bias. Direct observation in real-world settings provides a more objective assessment of behavior. In this context, CCTV-based observation offers a valuable opportunity to examine naturally occurring helmet-related behavior without disrupting routine traffic flow. Previous research has also shown that CCTV-supported monitoring can be integrated into traffic safety systems and may improve surveillance and enforcement efficiency [10].

Recent international research further demonstrates that helmet-related behavior varies across settings, user roles, and enforcement contexts. In Ho Chi Minh City, Vietnam, repeated cross-sectional observations documented consistently high observed helmet wearing but a marked decline in correct helmet use over time, underscoring the importance of distinguishing visible helmet use from correct protective use [16]. In Addis Ababa, Ethiopia, a six-year observational study found substantial differences between drivers and passengers and showed that correct helmet use was influenced by enforcement and road context [17]. In rural Ghana, recent evidence suggested that structural barriers, including the lack of an additional helmet for passengers, were important determinants of helmet use [18]. Taken together, these studies show that direct observation provides valuable insight into helmet-related behavior, while also highlighting that observed patterns are strongly shaped by context.

Although CCTV-based helmet monitoring has previously been reported in traffic safety research, most prior studies have focused on public roads or urban traffic settings rather than routine travel within a university environment [10,16,17]. The present study adds to the literature by providing event-based observational evidence from a Thai university campus and by describing visible helmet presence separately for riders and passengers, students and staff, and morning and evening commuting periods. In this way, the study contributes contextual public health evidence from a campus setting rather than proposing a new surveillance technology. To improve analytical clarity, the study was guided by the following research questions: (1) What is the level of visible helmet presence among motorcycle-user observation events at a university campus? (2) How does visible helmet presence vary by rider/passenger role, university affiliation, and time of day? (3) What patterns of daily variation are observable across the 7-day surveillance period?

### Conceptual Framework of the Study

The present study was guided by an observational public health framework in which motorcycle-user behavior within the university environment was examined through routine CCTV surveillance. Within this framework, visible helmet presence was defined as the primary observed outcome, while rider/passenger role, university affiliation, time of day, and day of observation were treated as contextual factors used to describe patterns in the observed events.

The purpose of this framework was not to test causal pathways or evaluate an automated detection system. Rather, it was used to organize real-world surveillance data in a way that could support campus road-safety planning and targeted health promotion. Accordingly, the study focused on identifying high-risk groups and time periods based on observed behavioral patterns in a natural university setting.

Conceptually, the framework links three related components. First, it reflects the campus traffic environment in which motorcycle riders and passengers enter and leave the university during routine daily activities. Second, it represents the systematic observation and classification process, in which CCTV footage was reviewed and coded according to predefined study variables. Third, it emphasizes the use of these observational data as public health evidence to inform campus safety policy, road-safety promotion, and future preventive interventions.

**Figure 1:**
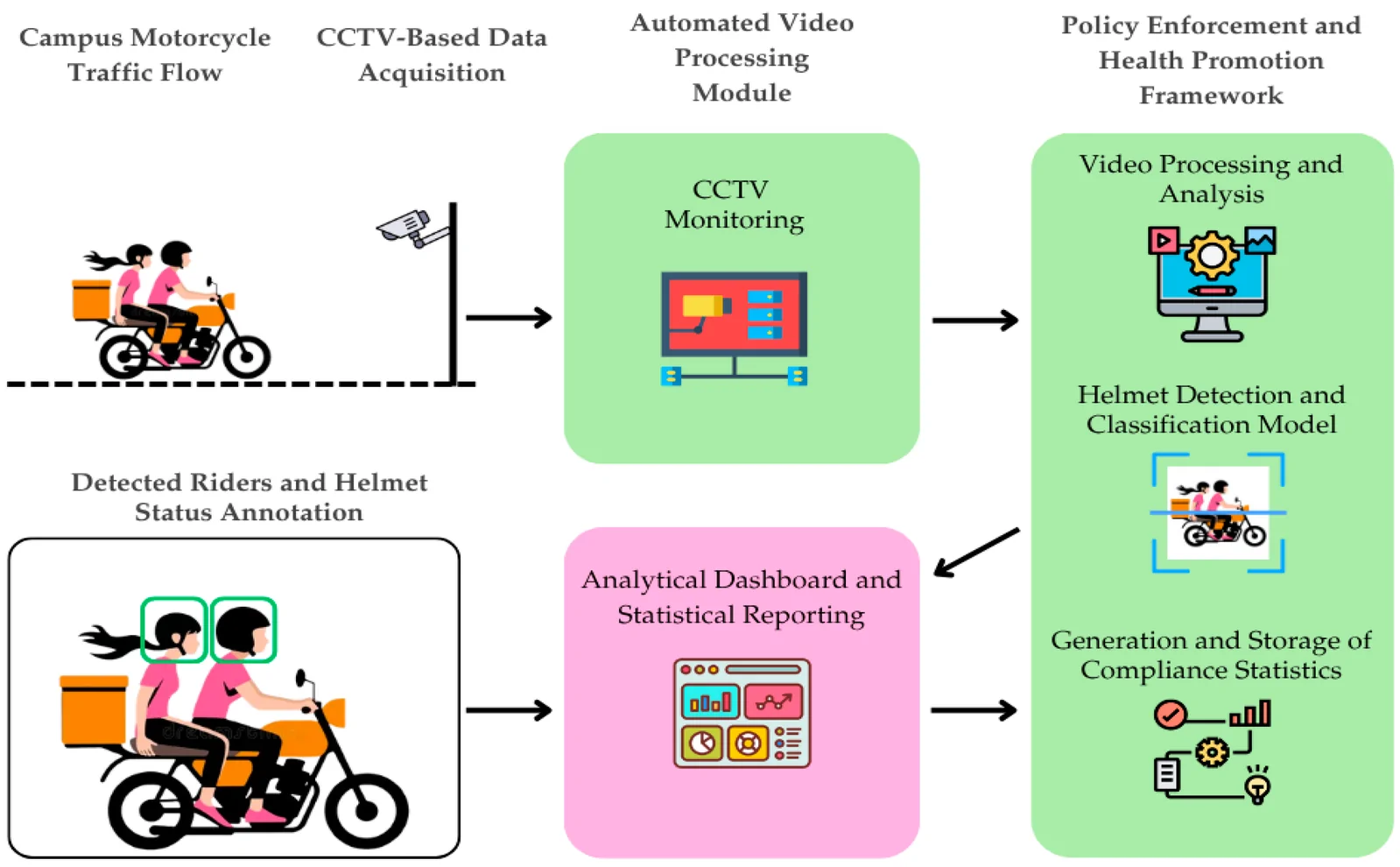
Analytical framework for CCTV-based observation of visible helmet presence in a university setting. Note. The framework illustrates how routine CCTV observation can be used to classify motorcycle-user observation events by role, affiliation, and time of day in order to generate descriptive evidence for campus road-safety planning.

Thus, the conceptual framework positions visible helmet presence not only as an individual safety-related behavior, but also as a measurable indicator of campus road-safety conditions. In this way, the framework supports the overall logic of the study by linking routine surveillance, structured observation, and evidence-informed action for injury prevention in the university setting (Figure 1).

## 2. Methods

### 2.1. Study Design and Setting

This study used a cross-sectional observational design based on retrospective review of CCTV footage collected during routine campus operations. The study was conducted at Rangsit University, a large private university in Thailand with substantial daily motorcycle traffic among students and university personnel. Two high-traffic observation points were selected: Gate 1, a major campus entry point, and Gate 2, a major campus exit point. These were separate physical campus access points. CCTV cameras are routinely installed at both gates for campus security and traffic monitoring.

Footage was reviewed over seven consecutive days during two peak commuting windows: 07:00–09:00 and 16:00–18:00. These periods were selected to capture routine inbound and outbound motorcycle traffic. The study took place in February 2026 during the second semester, coinciding with a regular teaching period.

Gate-specific analysis was not performed because the primary aim of the study was to describe overall campus commuting patterns across peak periods rather than compare directional traffic flows. This is acknowledged as a limitation. The primary outcome variable was visible helmet presence, coded as helmet visible or helmet not visible according to whether a helmet could be seen at the time the motorcycle passed the observation point. The study did not assess helmet quality, proper fastening, or whether the helmet was worn correctly.

### 2.2. Unit of Analysis

The unit of analysis was the motorcycle-user observation event rather than a confirmed unique individual. Accordingly, the same rider or passenger could potentially have been observed more than once across different gates, time periods, or days. The findings should therefore be interpreted as describing visible helmet presence across motorcycle-user observation events rather than estimating person-level prevalence among unique individuals.

### 2.3. Study Variables and Operational Definitions

A structured observation form was used to record variables visible in the CCTV footage. The primary outcome variable was visible helmet presence, coded as helmet visible or helmet not visible according to visible helmet use at the time the motorcycle passed the observation point. The study did not assess whether helmets were properly fastened, were of adequate quality, or were worn correctly.

Additional observed variables included role, classified as rider or passenger; university affiliation, classified as student or staff; time of observation, classified as morning (07:00–09:00) or evening (16:00–18:00); and day of observation, recorded as Day 1 to Day 7.

University affiliation was classified using visible contextual cues available in the footage, including student uniforms, staff attire, visible identification cards, and other campus-specific appearance indicators. When affiliation could not be determined with sufficient confidence, the event would have been classified as ambiguous and excluded from affiliation-stratified analysis. In the present dataset, no ambiguous cases were identified (*n* = 0). No personally identifiable information was recorded in the analytic dataset.

### 2.4. Data Collection Procedures

CCTV footage was reviewed systematically in 30-min intervals throughout each observation session. During each interval, motorcycle-user observation events were identified and coded using the structured observation form. Data extraction focused on visible helmet presence and the other predefined study variables.

Because the study relied on routinely collected surveillance footage, the observation process reflected natural traffic conditions within the campus environment. This approach allowed the research team to assess helmet-related behavior as it occurred in everyday university travel without direct interaction with riders or passengers and without altering the traffic environment.

Before formal data extraction, the coding team reviewed the operational definitions and conducted a pilot review of footage to improve consistency in classification decisions. Formal inter-rater reliability statistics were not available, and this is acknowledged as a limitation of the study.

### 2.5. Inclusion and Exclusion Criteria for Observation Events

Eligible observation events included motorcycles captured by CCTV at the selected campus gates during the predefined observation periods for which helmet use and rider/passenger status could be visually determined. Observation events were excluded when image quality was insufficient for coding, such as blurred footage, poor lighting, obstructed views, or incomplete visual capture of the motorcycle user.

### 2.6. Statistical Analysis

Data were analyzed using Microsoft Excel. Descriptive statistics were used to summarize visible helmet presence overall and by subgroup. Frequencies and percentages were calculated for the four principal analytic groups: student riders, student passengers, staff riders, and staff passengers. Visible helmet presence was also described according to time of day and day of observation.

In addition to descriptive statistics, chi-square tests were used to compare visible helmet presence across rider/passenger role, university affiliation, and time of day. For key proportions, 95% confidence intervals were calculated. Because the unit of analysis was the observation event rather than a unique individual, inferential findings were interpreted cautiously and as comparisons of event-based proportions rather than independent person-level observations.

### 2.7. Ethical Considerations

This study involved secondary analysis of CCTV recordings originally collected for routine campus safety monitoring. Ethical approval was granted by the Research Ethics Review Board of Rangsit University (COA No. RSUERB2025-235). The study posed minimal risk because no personal identifiable information was extracted, and all footage was reviewed in a secure environment by authorized personnel only. All procedures were conducted in accordance with institutional ethical requirements for observational research using routinely collected surveillance data.

## 3. Results

### 3.1. Overall Visible Helmet Presence

A total of 19,363 motorcycle-user observation events were recorded during the 7-day study period. Overall visible helmet presence was 18.8% (3646/19,363). Table 1 presents event-based visible helmet presence by user group.

Among student-rider observation events, visible helmet presence was 21.8% (1956/8985), whereas among student-passenger observation events it was 3.1% (230/7505). Among staff-rider observation events, visible helmet presence was 58.6% (1299/2217), and among staff-passenger observation events it was 24.5% (161/656). Across both affiliation groups, rider events showed higher visible helmet presence than passenger events, and staff events showed higher visible helmet presence than student events.

### 3.2. Subgroup and Time-of-Day Comparisons of Visible Helmet Presence

Marked differences in visible helmet presence were observed across the four analytic groups. Among students, rider observation events showed significantly higher visible helmet presence than passenger observation events (21.8% vs. 3.1%; χ^2^ = 1244.22, *p* < 0.001). Among staff, rider events also showed significantly higher visible helmet presence than passenger events (58.6% vs. 24.5%; χ^2^ = 234.83, *p* < 0.001).

Comparing affiliations within the same role, staff-rider events showed significantly higher visible helmet presence than student-rider events (58.6% vs. 21.8%; χ^2^ = 1169.68, *p* < 0.001), and staff-passenger events showed significantly higher visible helmet presence than student-passenger events (24.5% vs. 3.1%; χ^2^ = 610.08, *p* < 0.001).

In aggregated analyses, rider events overall showed higher visible helmet presence than passenger events overall (31.0% vs. 4.8%; χ^2^ = 1818.99, *p* < 0.001), and staff events overall showed higher visible helmet presence than student events overall (50.9% vs. 13.3%; χ^2^ = 2258.54, *p* < 0.001). These findings should be interpreted as event-based comparisons rather than person-level prevalence estimates (Table 2).

Visible helmet presence was also higher during the morning observation period than during the evening period across most analytic groups. Among student-rider events, visible helmet presence was significantly higher in the morning than in the evening (24.9% vs. 18.0%; χ^2^ = 62.74, *p* < 0.001). A similar pattern was observed among student-passenger events (3.9% vs. 1.8%; χ^2^ = 26.60, *p* < 0.001) and staff-rider events (61.3% vs. 54.6%; χ^2^ = 9.79, *p* = 0.002). Among staff-passenger events, morning visible helmet presence was also higher than evening visible helmet presence (27.6% vs. 21.1%), although this difference did not reach conventional statistical significance (χ^2^ = 3.71, *p* = 0.054). Overall, morning observation events showed significantly higher visible helmet presence than evening events (21.0% vs. 16.0%; χ^2^ = 77.59, *p* < 0.001).

### 3.3. Daily Variation

Daily variation in visible helmet presence across motorcycle-user observation events is presented in Table 3. Across Days 1 to 6, visible helmet presence remained consistently low, particularly among student-passenger events, which ranged from 1.9% to 3.7%. Among student-rider events, the corresponding values ranged from 12.7% to 24.1%. Staff-rider events consistently showed higher visible helmet presence than all other groups, ranging from 49.8% to 61.7% during Days 1 to 6, whereas staff-passenger events ranged from 9.1% to 27.4%.

Day 7 showed a marked increase in visible helmet presence across all groups, reaching 42.8% among student-rider events, 11.3% among student-passenger events, 75.8% among staff-rider events, and 40.5% among staff-passenger events. Day 7 fell on a weekend; however, the university remained active because some faculties and colleges continued teaching in special weekend programs, including selected master’s and doctoral courses. Therefore, the composition of campus motorcycle-user observation events on Day 7 may have differed from that of regular weekdays.

Because no direct contextual data on enforcement activity, weather conditions, or other situational factors were collected, the reason for this deviation could not be determined with certainty. Accordingly, Day 7 should be interpreted with caution. To examine its influence on the pooled estimate, a sensitivity analysis was performed excluding Day 7. This reduced the overall visible helmet presence estimate from 18.8% (3646/19,363) to 17.2% (3079/17,900), indicating that Day 7 materially influenced the overall result (Table 3).

## 4. Discussion

### 4.1. Principal Findings

This CCTV-based cross-sectional observational study found that visible helmet presence was low across motorcycle-user observation events at a Thai university campus. Overall visible helmet presence was 18.8%, with the lowest event-based estimate observed among student-passenger events and the highest among staff-rider events. Across both university affiliation groups, rider events consistently showed higher visible helmet presence than passenger events, and staff events consistently showed higher visible helmet presence than student events. Visible helmet presence was also higher during the morning commuting period than during the evening period across most analytic groups.

The very low visible helmet presence observed among student-passenger events is an important public health concern. Although passengers may be less likely than riders to carry helmets or may perceive short campus trips as low risk, the present study did not collect attitudinal or behavioral data to test these possibilities directly. Accordingly, such interpretations should be regarded as plausible contextual explanations rather than evidence-based causal conclusions.

The marked increase observed on Day 7 should also be interpreted cautiously. Day 7 fell on a weekend; however, the university remained active because some faculties and colleges continued teaching in special weekend programs, including selected master’s and doctoral courses. This suggests that the composition of motorcycle-user observation events on Day 7 may have differed from that of regular weekdays. Because no direct contextual data on enforcement activity, weather conditions, or other situational factors were collected, the reason for this deviation could not be determined with certainty.

Taken together, these findings suggest that low visible helmet presence was not uniformly distributed across campus traffic, but was concentrated in specific high-risk groups, particularly student passengers. From a public health perspective, this pattern is important because it identifies a subgroup that may require more targeted attention in campus safety promotion and injury-prevention planning.

### 4.2. Comparison with Existing Literature

The low visible helmet presence documented in the present study is broadly consistent with previous Thai literature showing that motorcycle helmet use remains suboptimal, particularly among passengers [5,12,13]. The rider-passenger gap observed in this study aligns with prior Thai evidence indicating that passengers are less likely than riders to wear helmets, even in settings where helmet laws are in place [5,12]. In this regard, the present findings reinforce the view that passengers remain a persistently under-protected group in motorcycle safety research and intervention.

The present findings are also broadly consistent with international observational studies showing that helmet-related behavior varies by setting, user role, and contextual factors. Studies from Vietnam [16] and Ethiopia [17] have shown that direct observation can identify meaningful differences in helmet-related behavior between user groups and over time, while research from Ghana [18] has highlighted structural barriers affecting passenger helmet use. In this context, the present study adds campus-specific, event-based evidence by showing differences in visible helmet presence according to rider/passenger role, university affiliation, and time of day in a university commuting environment (Table 4).

The values observed in this university setting also appear lower than estimates reported in some previous Thai surveys and self-reported studies. One likely explanation is methodology. Self-reported data may overestimate safety behavior because respondents may provide socially desirable answers or report what they believe they usually do rather than what they actually did in a specific event. By contrast, the present study captured naturally occurring behavior through routine CCTV observation, which may provide a more conservative but behaviorally grounded estimate of visible helmet presence during everyday campus travel [7,10].

The higher visible helmet presence observed among staff events compared with student events may reflect differences in age, risk perception, social responsibility, travel purpose, or awareness of legal and safety consequences. Previous studies suggest that helmet-related intention and safe riding behavior are influenced by attitudes, perceived behavioral control, subjective norms, and internal locus of control [1,14]. These factors may be more strongly established among staff than among younger university students, especially in routine short-distance commuting contexts where students may perceive lower vulnerability. However, because the present study did not collect explanatory variables, these interpretations should remain cautious.

Similarly, the descriptive pattern of higher morning values and lower evening values is noteworthy but cannot be explained definitively from the current data. The pattern may reflect differences in time pressure, fatigue, trip purpose, social context, or perceived visibility of monitoring across the day. Because such contextual variables were not measured, these possibilities should be treated as hypotheses for future research rather than firm conclusions.

### 4.3. Implications for Campus Health Promotion and Policy

The findings of this study have direct implications for campus road-safety policy and health promotion. First, universities should not assume that general traffic laws alone are sufficient to ensure consistent visible helmet presence within campus environments. The very low visible helmet presence observed among student-passenger events suggests the need for interventions that explicitly address this overlooked group rather than focusing only on riders. Educational strategies should therefore emphasize that passenger helmet use is equally important and legally relevant, even for short or familiar journeys [12,13].

Second, interventions should move beyond awareness campaigns alone and incorporate environmental and organizational support. Universities may consider visible gate-based messaging, campus helmet campaigns, peer-led communication, and practical measures such as helmet access or storage solutions. Because previous work has shown that CCTV-supported monitoring can influence helmet-related behavior in Thai traffic settings, campus surveillance data may also be useful as part of a broader safety feedback system when implemented with appropriate ethical safeguards [10].

Third, the present study supports the value of routine campus surveillance data as a tool for injury-prevention planning. CCTV systems already used for campus security can provide actionable behavioral indicators for health promotion, traffic management, and policy evaluation. In this sense, visible helmet presence may be viewed not only as an individual safety-related behavior, but also as an institutional road-safety indicator that can guide targeted intervention in high-risk groups and time periods.

### 4.4. Strengths and Limitations

This study has several strengths. It used unobtrusive CCTV-based observation to assess helmet-related behavior under real-world conditions, thereby reducing the likelihood of recall bias and social desirability bias associated with self-reported measures [7]. The study also included a large number of motorcycle-user observation events across seven consecutive days and two peak commuting periods, allowing visible helmet presence to be described by rider-passenger role, university affiliation, time of day, and day of observation. These features strengthen the practical relevance of the findings for campus safety planning.

Several limitations should also be acknowledged. First, the unit of analysis was the observation event rather than a unique individual, and the same person may therefore have been observed more than once across gates, time periods, or study days. The findings should accordingly be interpreted as event-based estimates rather than person-based prevalence estimates. Because repeated observations of the same individual were possible, inferential findings should be interpreted as comparisons of event-based proportions rather than independent person-level risks.

Second, the study was conducted at a single private university in Thailand. Campus traffic patterns, enforcement norms, commuting behavior, and institutional culture may differ across universities and urban settings; therefore, the findings should be interpreted cautiously outside the present context. Future multi-campus studies are needed to examine whether similar patterns are observed elsewhere.

Third, classification of student and staff status relied on visible contextual cues and may therefore have introduced misclassification bias. Although operational coding rules were used and no ambiguous affiliation cases were identified in the present dataset, affiliation could not be verified directly from the footage. In addition, formal inter-rater reliability statistics were not available, which should be considered when interpreting the consistency of manual classification from CCTV footage.

Fourth, the study did not collect attitudinal, behavioral, or enforcement-related variables that could explain why visible helmet presence differed between groups or time periods. Fifth, the study assessed visible helmet presence only and did not evaluate helmet quality, proper fastening, or whether the helmet was worn correctly [3,4]. Visible helmet presence may therefore overestimate actual protective behavior. Finally, because no contextual variables were recorded beyond the observation form, the reasons for daily fluctuation, including the higher values observed on Day 7, could not be determined with certainty.

## 5. Conclusions

This CCTV-based cross-sectional observational study found that visible helmet presence was low across motorcycle-user observation events at a Thai university campus. Overall visible helmet presence was 18.8%, with the lowest event-based estimate observed among student-passenger events and the highest among staff-rider events. Across both university affiliation groups, rider events showed higher visible helmet presence than passenger events, and staff events showed higher visible helmet presence than student events. Visible helmet presence was also generally higher during morning observation periods than during evening periods.

These findings highlight an important campus road-safety gap and suggest that university motorcycle travel should not be assumed to be low risk simply because it occurs in a familiar or short-distance environment. In particular, the very low visible helmet presence among passenger events indicates the need for more targeted and inclusive safety strategies that address both riders and passengers.

The study also demonstrates the value of routine CCTV data for generating real-world behavioral evidence in campus safety research. When used ethically and appropriately, such data can support injury-prevention planning, identify high-risk groups and time periods, and inform targeted interventions. However, the findings should be interpreted as event-based estimates of visible helmet presence rather than person-based prevalence or verified protective helmet use. Future multi-campus studies should examine whether similar patterns are observed across other educational settings and should incorporate broader contextual and behavioral variables to strengthen interpretation.

## Figures and Tables

**Table 1:** Visible helmet presence by user group.

User Group	Helmet Visible (*n*)	Total Observed (*n*)	Visible Helmet Presence (%)	95% CI
Student riders	1956	8985	21.8	20.9–22.6
Student passengers	230	7505	3.1	2.7–3.5
Staff riders	1299	2217	58.6	56.5–60.6
Staff passengers	161	656	24.5	21.4–28.0
Overall	3646	19,363	18.8	18.3–19.4

**Table 2:** Visible helmet presence by time of day.

User Group	Morning, *n*/*N* (%)	95% CI	Evening, *n*/*N* (%)	95% CI	χ^2^	*p*-Value
Student riders	1224/ 4914 (24.9)	23.7–26.1	732/ 4071 (18.0)	16.8–19.2	62.74	<0.001
Student passengers	173/ 4408 (3.9)	3.4–4.5	57/ 3097 (1.8)	1.4–2.4	26.60	<0.001
Staff riders	816/ 1332 (61.3)	58.6–63.8	483/ 885 (54.6)	51.3–57.8	9.79	0.002
Staff passengers	96/ 348 (27.6)	23.2–32.5	65/ 308 (21.1)	16.9–26.0	3.71	0.054
Overall	2309/ 11,002 (21.0)	20.2–21.8	1337/ 8361 (16.0)	15.2–16.8	77.59	<0.001

**Table 3:** Daily visible helmet presence by user group, presented as *n*/*N* (%).

Day	Student Riders *n*/*N* (%)	Student Passengers *n*/*N* (%)	Staff Riders *n*/*N* (%)	Staff Passengers *n*/*N* (%)
Day 1	350/1743 (20.1)	38/1447 (2.6)	255/413 (61.7)	21/97 (21.6)
Day 2	456/2395 (19.0)	41/1779 (2.3)	223/423 (52.7)	37/135 (27.4)
Day 3	313/1347 (23.2)	47/1268 (3.7)	164/278 (59.0)	21/96 (21.9)
Day 4	291/1452 (20.0)	24/1290 (1.9)	194/336 (57.7)	9/61 (14.8)
Day 5	229/952 (24.1)	19/862 (2.2)	152/287 (53.0)	16/75 (21.3)
Day 6	64/505 (12.7)	8/390 (2.1)	101/203 (49.8)	6/66 (9.1)
Day 7	253/591 (42.8)	53/469 (11.3)	210/277 (75.8)	51/126 (40.5)

Note. Day 7 showed a marked increase in visible helmet presence across all analytic groups and should be interpreted with caution. Although Day 7 fell on a weekend, the university remained active because some faculties and colleges continued teaching in special weekend programs, including selected master’s and doctoral courses. Therefore, the composition of motorcycle-user observation events on Day 7 may have differed from that of regular weekdays. Excluding Day 7, the overall estimate changed from 18.8% (3646/19,363) to 17.2% (3079/17,900).

**Table 4:** Comparison of the present study with selected previous research.

Study	Setting	Design/Approach	Main Focus	Contribution Relative to the Present Study
Li et al. [16]	Ho Chi Minh City, Vietnam	Repeated cross-sectional observational study	Observed helmet use and correct helmet use over time in an urban traffic setting	Demonstrates the value of large-scale direct observation, but focuses on urban road traffic rather than a university environment
Shifaw et al. [17]	Addis Ababa, Ethiopia	Six-year observational study	Driver-passenger differences and contextual factors associated with correct helmet use	Shows the importance of long-term surveillance and enforcement context, but not a campus-based commuting setting
Morgan [18]	Rural Ghana	Cross-sectional study	Contextual and behavioral determinants of helmet use, including lack of passenger helmets	Highlights structural barriers relevant to passenger use, supporting interpretation of low passenger values in the present study
Present study	Rangsit University, Thailand	CCTV-based cross-sectional observational study	Visible helmet presence by rider/passenger role, university affiliation, and time of day	Adds event-based evidence from a university campus environment and identifies subgroup and temporal patterns relevant to campus road-safety planning

## Data Availability

The data that support the findings of this study are available from Rangsit University upon reasonable request and with institutional permission, subject to restrictions related to security and privacy.
